# Characteristics and outcome after out-of-hospital cardiac arrest with the emphasis on workplaces: an observational study from the Swedish Registry of Cardiopulmonary Resuscitation

**DOI:** 10.1016/j.resplu.2021.100090

**Published:** 2021-02-18

**Authors:** Helene Bylow, Araz Rawshani, Andreas Claesson, Margret Lepp, Johan Herlitz

**Affiliations:** a Department of Molecular and Clinical Medicine, Institute of Medicine, Sahlgrenska Academy, University of Gothenburg, Gothenburg, Sweden; b Department of Medicine, Centre for Resuscitation Science, Karolinska Institute, Stockholm, Sweden; c Institute of Health and Care Sciences, Sahlgrenska Academy, University of Gothenburg, Gothenburg, Sweden; d Østfold University College, Halden, Norway; e School of Nursing and Midwifery, Griffith University, Brisbane, Australia; f Centre of Registers Västra Götaland, Gothenburg, Sweden; g Prehospen-Centre of Prehospital Research, Faculty of Caring Science, Work Life and Social Welfare, University of Borås, Borås, Sweden

**Keywords:** Out-of-hospital cardiac arrest, Register, Utstein style, Location, Workplace, Public place

## Abstract

**Background:**

Characteristics and outcome in out-of-hospital cardiac arrest (OHCA) occurring at workplaces is sparsely studied.

**Aim:**

To describe (1) the characteristics and 30-day survival of OHCAs occurring at workplaces in comparison to OHCAs at other places and (2) factors associated with survival after OHCAs at workplaces.

**Methods:**

Data on OHCAs were obtained from the Swedish Registry of Cardiopulmonary Resuscitation from 1 January 2008 to 31 December 2018. Characteristics and factors associated with survival were analysed with emphasis on the location of OHCAs.

**Results:**

Among 47,685 OHCAs, 529 cases (1%) occurred at workplaces. Overall, in the fully adjusted model, all locations of OHCA, with the exception of crowded public places, displayed significantly lower probability of survival than workplaces. Exhibiting a shockable rhythm was the strongest predictor of survival among patients with OHCAs at workplaces; odds ratio (95% CI) 5.80 (2.92–12.31). Odds ratio for survival for women was 2.08 (95% CI 1.07–4.03), compared with men. At workplaces other than private offices, odds ratio for survival was 0.41 (95% CI 0.16–0.95) for cases who did not receive bystander CPR, as compared to those who did receive CPR. Among patients who were found in a shockable rhythm were 23% defibrillated before arrival of ambulance, which was more frequent than in any other location.

**Conclusion:**

Out-of-hospital cardiac arrest occurring at workplaces and crowded public places display the highest probability of survival, as compared with other places outside hospital. An initial shockable cardiac rhythm was the strongest predictor of survival for OHCA at workplaces.

## Introduction

Out-of-hospital cardiac arrest (OHCA) in adults is a leading cause of death and needs to be thoroughly elucidated.^1^, ^2^, ^3^, ^4^, ^5^, ^6^ Treatment with cardiopulmonary resuscitation (CPR) and automated external defibrillation (AED) has been shown to increase survival, but the survival rate after OHCA still remains poor.^3^, ^7^ The global annual incidence of OHCA has been estimated at 30–97 per 100,000 person-years^7^ and the 30-day survival rate for OHCAs has been reported to be 11%,^8^ thereby ranging between 3% and 20%.^7^ Factors associated with higher survival are described as the OHCA being witnessed, if the patient received early CPR, had a detectable shockable rhythm and with the early use of a publicly accessible defibrillator (PAD).^8^, ^9^, ^10^, ^11^, ^12^

In Sweden, both OHCA and in-hospital cardiac arrests where resuscitation is attempted are reported to the Swedish Registry of Cardiopulmonary Resuscitation (SRCR).^11^ The number and availability of public AEDs is reported by the owner to the Swedish AED Registry and 43% are reported to be placed at workplaces.^13^

The incidence of OHCAs at workplaces is reported to be as low as 0.3%–4.7% of all OHCAs.^14^ The long-term survival has been reported to range from 5.8%–13.4% and recently to 27.5% which is relatively high if compared with all OHCAs.^14^, ^15^, ^16^, ^17^ The characteristics of patients with OHCAs at workplaces differ from those of general OHCAs by being younger, more frequently males, having a presumed cardiac aetiology, witnessed cases and as having received bystander CPR more frequently.^14^, ^16^, ^17^

Hypothetically, the chance of surviving an OHCA at a workplace location should be higher compared with OHCAs at other places.^14^, ^18^ The reason is that preparedness for an emergency situation at workplaces is regulated by the Work Environment Act and in Sweden a sufficient number of employees must have knowledge of basic life support (BLS).^19^ The aim of this study was therefore to describe (1) the characteristics and 30-day survival of OHCAs occurring at workplaces in comparison to OHCAs at other places and (2) factors associated with survival after OHCAs at workplaces.

## Methods

### Study design

This is a nationwide observational study based on cases enrolled between 2008 and 2018 in the SRCR which covers the entire population.^20^, ^21^ The study was conducted in Sweden with a total population ranging from nine million inhabitants in 2008 to 10 million in 2018.^22^ The reporting is mainly according to the Utstein style.^5^, ^6^, ^23^

### Data source

The SRCR is a national quality registry founded in 1990. All OHCAs which take place outside hospital where resuscitation is attempted by a bystander, first responder or EMS are reported and registered prospectively to the registry by the EMS personnel. Level of ascertainment is scrutinized by retrospective reassessments of EMS records. All EMS systems in Sweden report to the registry.^20^ The delays are estimated by the bystander, the EMS dispatcher and the EMS personnel. The delay to CPR and defibrillation is calculated from the collapse of the OHCA patient. Data were available from 1 January 2008 to 31 December 2018, except for the variable “defibrillated before EMS arrival”, which was available from 2009. The register is validated on a regular basis and is more thoroughly described elsewhere.^20^, ^24^

### Study population

The inclusion criteria were patients 18 years of age or older with an OHCA where resuscitation was attempted and cases with complete vital data. The exclusion criteria were patients younger than 18 years of age and cases with missing data. Due to the register-based design with retrospective analysis, informed consent was waived. However, the majority of patients who survive to three months are informed that they have been reported to the registry and are given the opportunity to withdraw from the registry, but there was no patient who withdrew their registration.

### Definitions

In this study, the definitions of the categories which were compared are by the location of the OHCA based on the SRCR.^25^ The location at a workplace is the reference. The definition of a workplace in the SRCR is the place where you work and includes all types of office and industrial workplaces except for the private office which is categorised separately. In this study we have included private office in the category of a workplace. The categories defined by the location of OHCA are as follows: (a) workplace: workplace, occupational, office, factory, industry, industrial business, public business, industrial building, work, small office, public office, construction sites and private office; (b) EMS-witnessed cases in the ambulance; (c) crowded public place such as shopping centre, public building, educational institution, bathing place, airport, street, highway, market, recreation ground, church, amusement field, sports arena, railway station and watercourse; (d) healthcare facilities outside hospital such as nursing home, primary care centre, dental clinic, hospital without acute care; e) home or in a residential setting; (f) hotel room; (g) unspecified non-public places; and (h) unspecified public places.

### Ethics

The Swedish Ethical Review Authority approved the ethical application on 28 October 2019 (2019-04066).

### Outcome

The primary outcome was defined as survival to 30 days (%) for EMS-treated cases of OHCA and factors that were independently associated with 30-day survival after OHCA.

### Statistics

Baseline characteristics are presented with means, medians and proportions, with the appropriate measurement of dispersion. Hypothesis testing was not performed for baseline characteristics.^26^ Time to CPR and time to defibrillation were compared across all locations using medians. Adjusted 30-day survival was examined using logistic regression. The predictor of main interest was the location of the cardiac arrest; adjustment was made for age, sex and calendar year. We computed one regression model for the entire study population and separate models for patients with a shockable and a non-shockable rhythm. We also used logistic regression to obtain odds ratios for 30-day survival in a model with additional covariates, namely witnessed arrest, bystander CPR, defibrillation, initial rhythm, use of adrenaline and cause of arrest. The analyses were performed by using R, version 4.0.1 (The R Project for Statistical Computing, https://www.r-project.org/).

## Results

The SRCR database with OHCA cases from 2008 to 2018 contained 54,189 patients. After exclusion of cases with incomplete data and patients younger than 18 years of age a total of 47,685 patients were included (Appendix, Table A1). Of these, 529 patients (1.1%) suffered an OHCA at workplaces (a). In this study, these patients are described in relation to patients with OHCAs at other places, i.e. (b) EMS-witnessed cases in the ambulance (n = 2,312, 4.8%), (c) at crowded public places (n = 5,844, 12.2%), (d) in healthcare facilities outside hospital (n = 1,574, 3.3%), (e) at home or in residential settings (n = 33,724, 70.7%), (f) in hotel rooms (n = 75, 0.2%), (g) at unspecified non-public places (n = 2,074, 4.3%) and (h) at unspecified public places (n = 1,553, 3.2%).

## Baseline characteristics

In what follows, patients who suffered OHCAs at workplaces are compared with patients who suffered OHCAs at other places. Patients who suffered an OHCA at workplaces had a mean age of 56 years, a younger mean age than in all the other groups (Table 1). The proportion of women was 15%, which was lower compared with all the other groups. When witnessed, the cases were witnessed by a bystander in 90% and in 91% at crowded public places. CPR was performed before the arrival of the EMS in 80%, which was higher than in all the other places. The first recorded rhythm was shockable in 47% and the patients were defibrillated in 61% of all cases, both of which were higher figures than in all the other groups. A presumed cardiac aetiology was found in 69% of victims at workplaces, which was the highest figure compared with the other categories. Chest compression-only CPR was performed in 50% of the cases where CPR was performed before the arrival of the EMS. The patients with OHCAs at workplaces, who were found in a shockable rhythm, were defibrillated before the arrival of the EMS in 23% of cases. This figure should be compared with 17% at crowded public places, nine per cent in healthcare facilities outside hospital, six per cent at home or in residential settings, 13% in hotel rooms, 15% at unspecified non-public places and 14% at unspecified public places. This figure was higher than at all other places.

**Table 1:** Baseline characteristics.

	Workplace, private office	Ambulance-witnessed by EMS	Crowded public place	Health care facility	Home, residential setting	Hotel-room	Un-specified non-public place	Un- specified public place
Total, n	529	2312	5844	1574	33,724	75	2074	1553
Age, years mean (SD)	55.9 (12.5)	72.0 (13.7)	64.4 (16.5)	75.2 (16.1)	70.4 (15.7)	62.2 (17.9)	61.1 (17.7)	65.8 (15.8)
Sex, female, n (%)	80 (15.1)	895 (38.7)	1099 (18.8)	718 (45.6)	12,412 (36.8)	16 (21.3)	440 (21.2)	310 (20.0)
Witnessed arrest, n (%)	360 (69.4)	2299 (99.7)	4042 (71.6)	1181 (76.4)	20,659 (62.6)	45 (60.8)	1260 (62.1)	1032 (68.7)
Witnessed arrest by a bystander, n (%)	321 (90.2)	NA	3639 (90.8)	953 (81.7)	16,779 (82.7)	31 (68.9)	1104 (88.5)	898 (87.6)
No bystander CPR, n (%)	105 (20.1)	NA	1542 (26.6)	429 (27.9)	13,077 (39.9)	19 (29.2)	557 (27.0)	458 (29.9)
Initial rhythm, VF/VT, shockable, n (%)	237 (46.6)	682 (32.5)	2276 (40.5)	173 (11.6)	5849 (17.9)	16 (22.5)	573 (28.7)	482 (32.2)
Defibrillation, n (%)	312 (60.9)	966 (42.9)	2899 (50.9)	310 (20.8)	10,176 (31.4)	26 (40.6)	831 (40.2)	670 (44.4)
Adrenaline not given, n (%)	104 (20.0)	1080 (47.8)	1526 (26.4)	360 (23.1)	5748 (17.2)	21 (28.0)	426 (20.6)	337 (21.9)
Cause of arrest, aetiology, n (%)								
Cardiac	343 (68.5)	1490 (66.6)	3635 (65.5)	871 (58.0)	20,453 (64.3)	46 (67.6)	1122 (56.1)	950 (64.5)
Accident	44 (8.8)	30 (1.3)	465 (8.4)	9 (0.6)	254 (0.8)	1 (1.5)	105 (5.3)	106 (7.2)
Drowning	0 (0.0)	0 (0.0)	283 (5.1)	1 (0.1)	35 (0.1)	0 (0.0)	29 (1.5)	18 (1.2)
Other	94 (18.8)	471 (21.1)	832 (15.0)	341 (22.7)	6641 (20.9)	8 (11.8)	435 (21.8)	245 (16.6)
Overdose	2 (0.4)	15 (0.7)	89 (1.6)	33 (2.2)	941 (3.0)	8 (11.8)	140 (7.0)	49 (3.3)
Pulmonary disease	7 (1.4)	205 (9.2)	76 (1.4)	110 (7.3)	1956 (6.2)	2 (2.9)	47 (2.4)	23 (1.6)
Suffocation	4 (0.8)	20 (0.9)	52 (0.9)	105 (7.0)	854 (2.7)	2 (2.9)	37 (1.9)	41 (2.8)
Suicide	7 (1.4)	6 (0.3)	118 (2.1)	31 (2.1)	665 (2.1)	1 (1.5)	84 (4.2)	41 (2.8)
Mode of bystander CPR, n (%)								
Compression and ventilation, n (%)	83 (49.1)	NA	766 (48.4)	308 (56.8)	2814 (37.3)	8 (42.1)	244 (46.7)	184 (46.5)
Chest compression-only, n (%)	84 (49.7)	NA	801 (50.6)	227 (41.9)	4601 (60.9)	11 (57.9)	276 (52.8)	206 (52.0)
Ventilation, only, n (%)	1 (0.6)	NA	9 (0.6)	2 (0.4)	50 (0.7)	0 (0.0)	1 (0.2)	3 (0.8)
Time from collapse to alarm, minutes, median [IQR]^a^	2.00 [1.00, 3.00]	NA	2.00 [1.00, 3.00]	2.00 [1.00, 5.00]	2.00 [1.00, 5.00]	2.00 [2.00, 4.75]	2.00 [1.00, 5.00]	2.00 [1.00, 4.00]
Time from collapse to CPR, minutes, median [IQR]^a^	1.00 [0.00, 5.00]	NA	2.00 [0.00, 5.00]	1.00 [0.00, 5.00]	5.00 [1.00, 10.00]	5.00 [2.00, 10.00]	2.00 [0.00, 7.00]	2.00 [0.00, 6.00]
Time from collapse to defibrillation, minutes, median [IQR]^a^	11.00 [8.00, 17.00]	NA	12.00 [8.00, 18.00]	14.00 [8.00, 22.00]	17.00 [12.00, 25.00]	17.50 [10.00, 20.50]	15.00 [9.00, 23.00]	13.00 [9.00, 20.00]
Time from arrival of call to EMS dispatch, minutes, median [IQR]^a^	1.00 [0.00, 1.00]	NA	1.00 [0.00, 1.00]	1.00 [0.00, 1.00]	1.00 [0.00, 2.00]	0.00 [0.00, 1.00]	1.00 [0.00, 1.00]	1.00 [0.00, 2.00]
Time from dispatch to EMS arrival, minutes, median [IQR]^b^	8.00 [5.00, 12.00]	NA	8.00 [5.00, 13.00]	9.00 [6.00, 13.00]	10.00 [7.00, 16.00]	8.50 [5.00, 18.00]	10.00 [6.00, 16.00]	8.00 [5.00, 15.00]
Defibrillated before EMS, arrival, n (%)^c^	62 (22.5)	NA	568 (17.2)	76 (9.0)	953 (6.3)	4 (13.3)	157 (15.2)	115 (14.1)

EMS, emergency medical service; SD, standard deviation; CPR, cardiopulmonary resuscitation; VF, ventricular fibrillation; VT, ventricular tachycardia; IQR, interquartile range; NA, not applicable.

a Only includes bystander witnessed cases.

b Does not include ambulance-witnessed cases.

c Does not include ambulance-witnessed cases and data were only available in 2009–2018.

## Delays

**Fig. 1:**
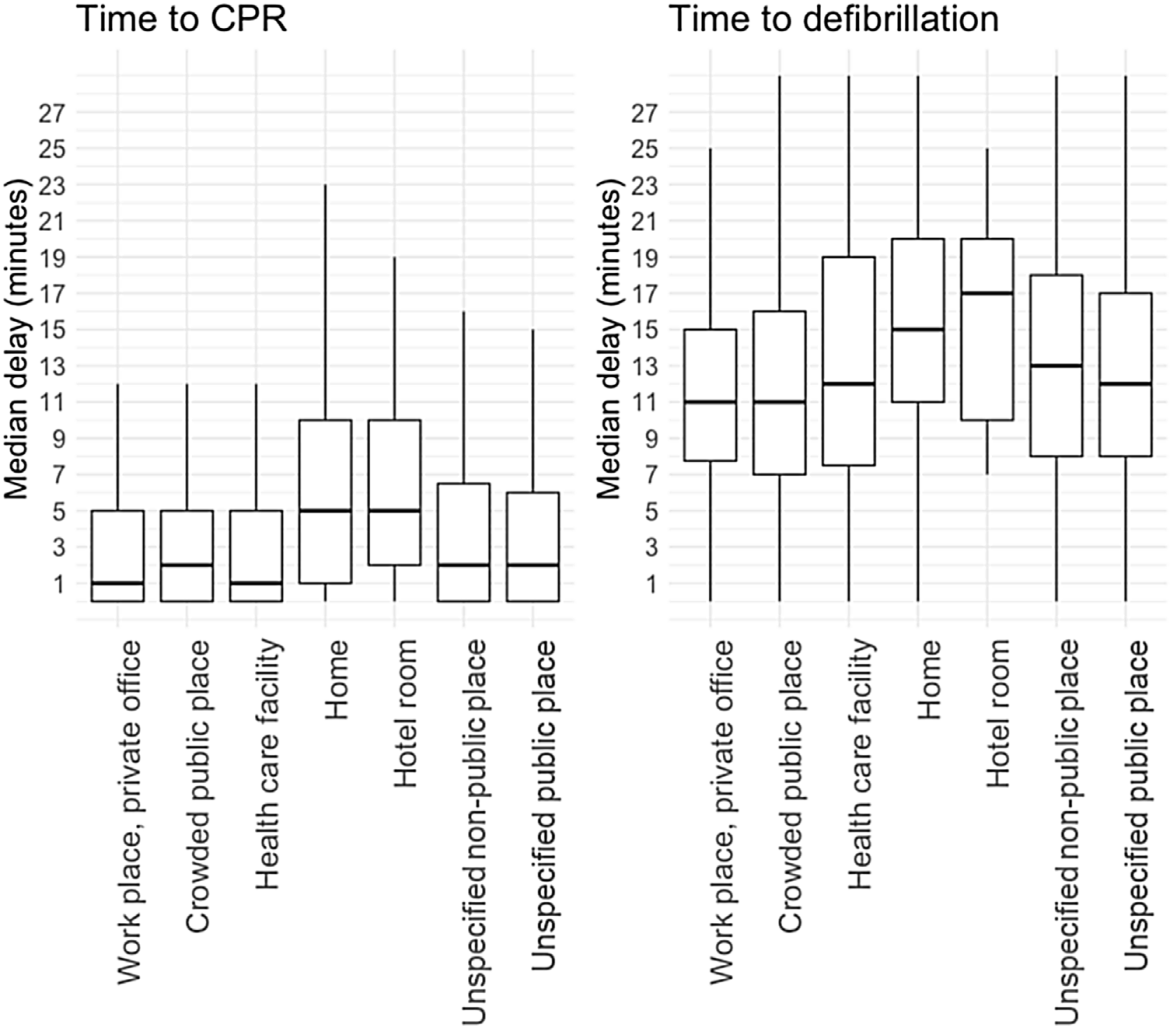
Delays from collapse to cardiopulmonary resuscitation and defibrillation. Delays from collapse to the start of cardiopulmonary resuscitation (CPR) and delays from collapse to defibrillation for bystander-witnessed out-of-hospital cardiac arrest (OHCA). Only bystander-witnessed OHCA included. Each boxplot represents each location of OHCA. Data is presented as the median delay in minutes (the second quartile, i.e. the 50th percentile), and the first and third quartile with the whiskers as the minimum and the maximum value.

The median delay from collapse due to an OHCA at workplaces to calling for an ambulance was two minutes, which was the same compared with other places (Table 1). The median delay from collapse to the start of CPR at workplaces was one minute, which was the same as at healthcare facilities but shorter than in the other places. The median delay from collapse to defibrillation among patients who were found in a shockable rhythm at workplaces was 11 min, which was shorter than in all the other categories of places. The median delay from dispatch to arrival of EMS at workplaces was eight minutes, which was similar to crowded public places and unspecified public places. When the OHCA was witnessed by a bystander, the delay to the start of CPR was one minute which was similar to healthcare facility and the delay to defibrillation was 11 min which was similar to crowded public places (Fig. 1).

## Survival to 30 days

The overall 30-day survival among patients with an OHCA at a workplace was 30% (n = 157), which was higher than at all the other places. The 30-day survival was 28% for cases which occurred in the ambulance, 23% at crowded public places, 9% in healthcare facilities outside hospital, 7% at home or in residential settings, 11% in hotel rooms, 16% at unspecified non-public places and 17% at unspecified public places.

**Fig. 2:**
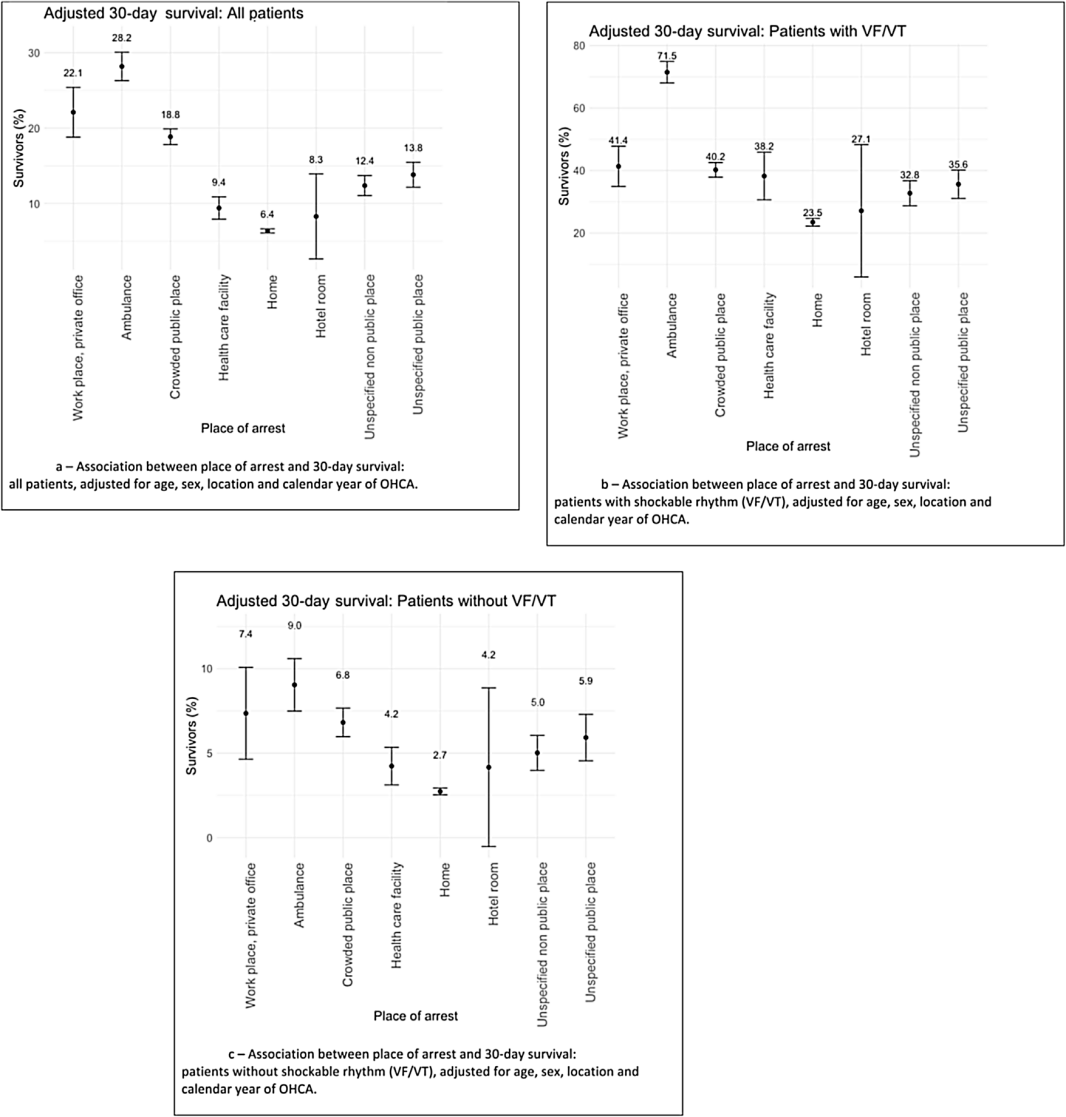
Association between place of arrest and 30-day survival. Adjusted odds ratio with 95% confidence interval for 30-day survival in relation to out-of-hospital cardiac arrest (OHCA) at workplaces: (a) Association between place of arrest and 30-day survival: all patients, adjusted for age, sex, location and calendar year of OHCA; (b) Association between place of arrest and 30-day survival: patients with a shockable rhythm (VF/VT), adjusted for age, sex, location and calendar year of OHCA; (c) Association between place of arrest and 30-day survival: patients without a shockable rhythm (VF/VT), adjusted for age, sex, location and calendar year of OHCA.

When adjusted for age, sex, place and calendar year of OHCA, the 30-day survival was lower than if witnessed by the EMS in the ambulance but higher than that among patients with an OHCA at other places (Fig. 2a). Among patients with a shockable rhythm (Fig. 2b) and with a non-shockable rhythm (Fig. 2c), the adjusted survival at workplaces was still lower than if the OHCA was witnessed by the EMS in the ambulance, but higher than in the other categories of different places.

**Fig. 3:**
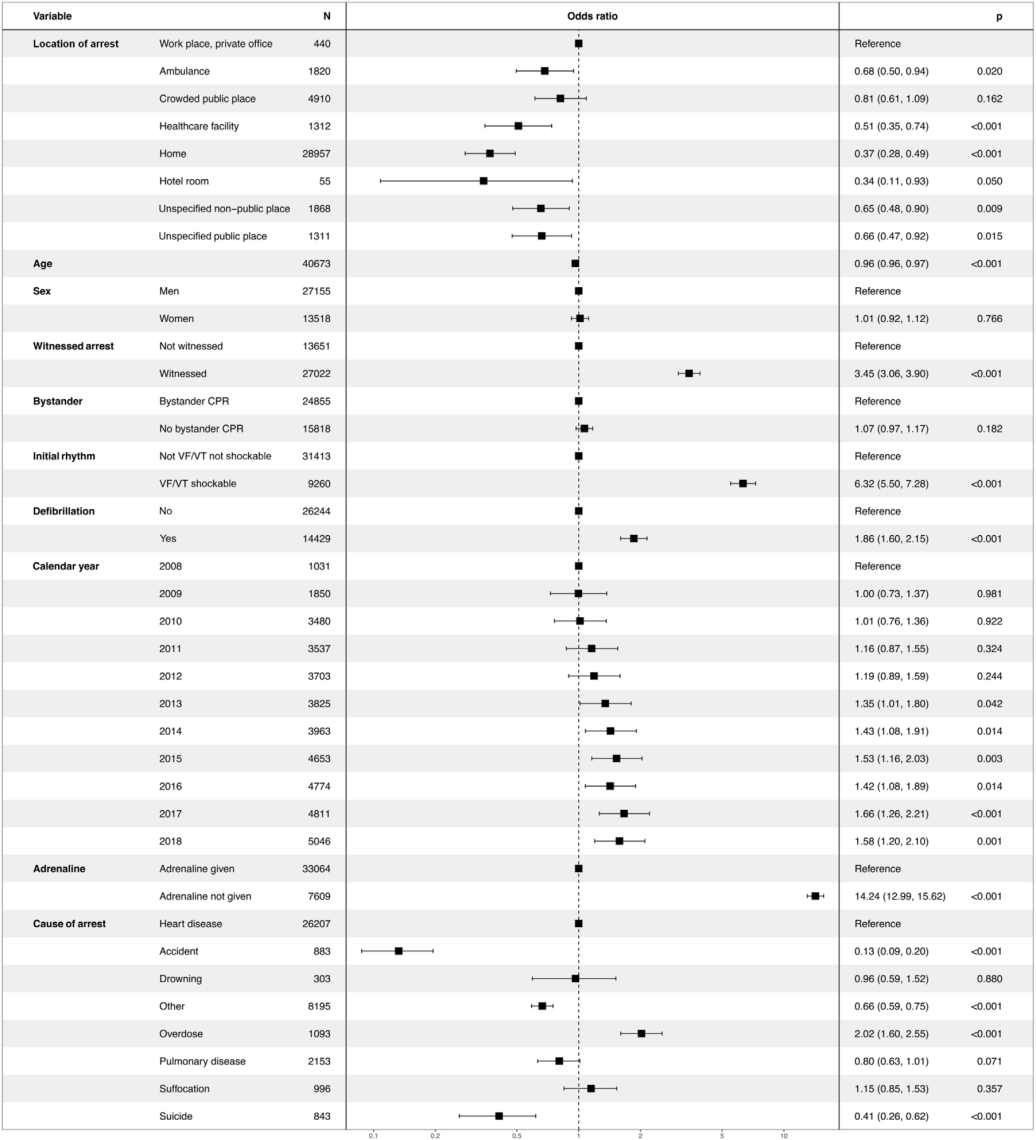
Factors associated with 30-day survival after out-of-hospital cardiac arrest (95% confidence interval). Factors described with odds ratio (95% confidence interval, CI, and p-value < 0.05 considered as significant), associated with 30-day survival after out-of-hospital cardiac arrest (OHCA), all locations of arrest included. Adjusted 30-day survival related to factors at resuscitation with OHCA at workplaces as a reference.

Fig. 3 shows the association between ten predictors (fully adjusted model) and 30-day survival in OHCA. All locations of OHCA, except for crowded public places, displayed significantly lower probability of survival than workplaces. Compared with workplaces the OR for ambulance was 0.68 (95% CI 0.50−0.94), crowded public place 0.81 (95% CI 0.61–1.09), healthcare facility 0.51 (95% CI 0.35−0.74), home 0.37 (95% CI 0.28−0.49), hotel room 0.34 (95% CI 0.11−0.93), unspecified non-public place (95% CI 0.48−0.90) and for unspecified public place the OR was 0.66 (95% CI 0.47−0.92).

**Fig. 4:**
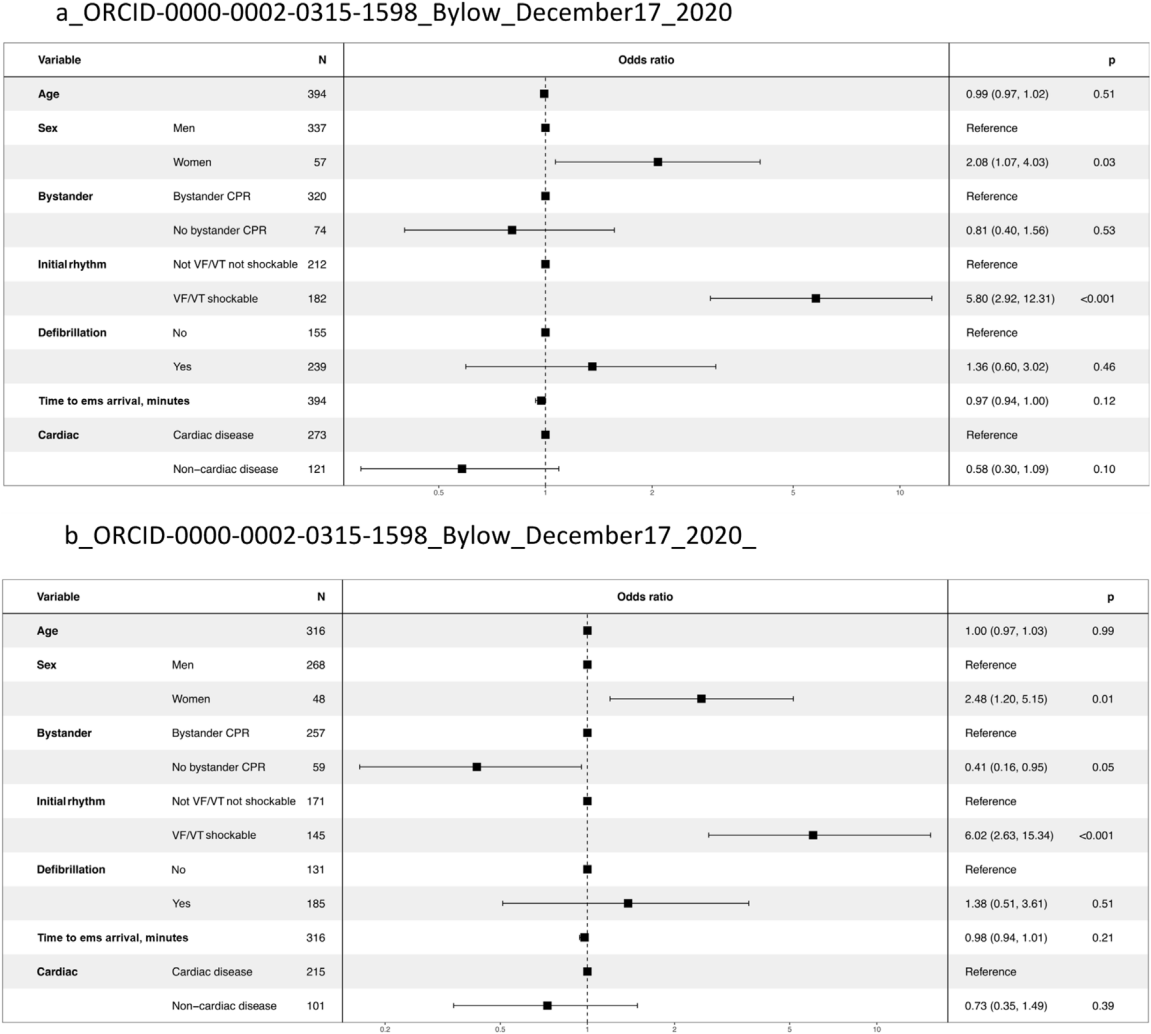
Factors associated with 30-day survival after out-of-hospital cardiac arrest at workplaces (95% confidence interval). (a) Factors described with odds ratio (95% confidence interval, CI, and p-value < 0.05 considered as significant), associated with 30-day survival after out-of-hospital cardiac arrest (OHCA) at workplaces; (b) Factors described with odds ratio (95% confidence interval, CI, and p-value < 0.05 considered as significant), associated with 30-day survival after OHCA at workplaces when private office was excluded from the location at workplaces.

Among patients with OHCAs at workplaces (Fig. 4a), exhibiting a shockable rhythm was the strongest predictor of survival to 30 days, with an OR of 5.80 (95% CI 2.92–12.31). Furthermore, female gender tended to be associated with an increased chance of survival with an OR of 2.08 (95% CI 1.07–4.03), compared with men. Finally, when private offices were excluded from the category workplaces (Fig. 4b), OR for survival was 0.41 (95% CI 0.16–0.95) for cases who did not receive bystander CPR, as compared with those who did receive bystander CPR.

## Aspects of time

**Fig. 5:**
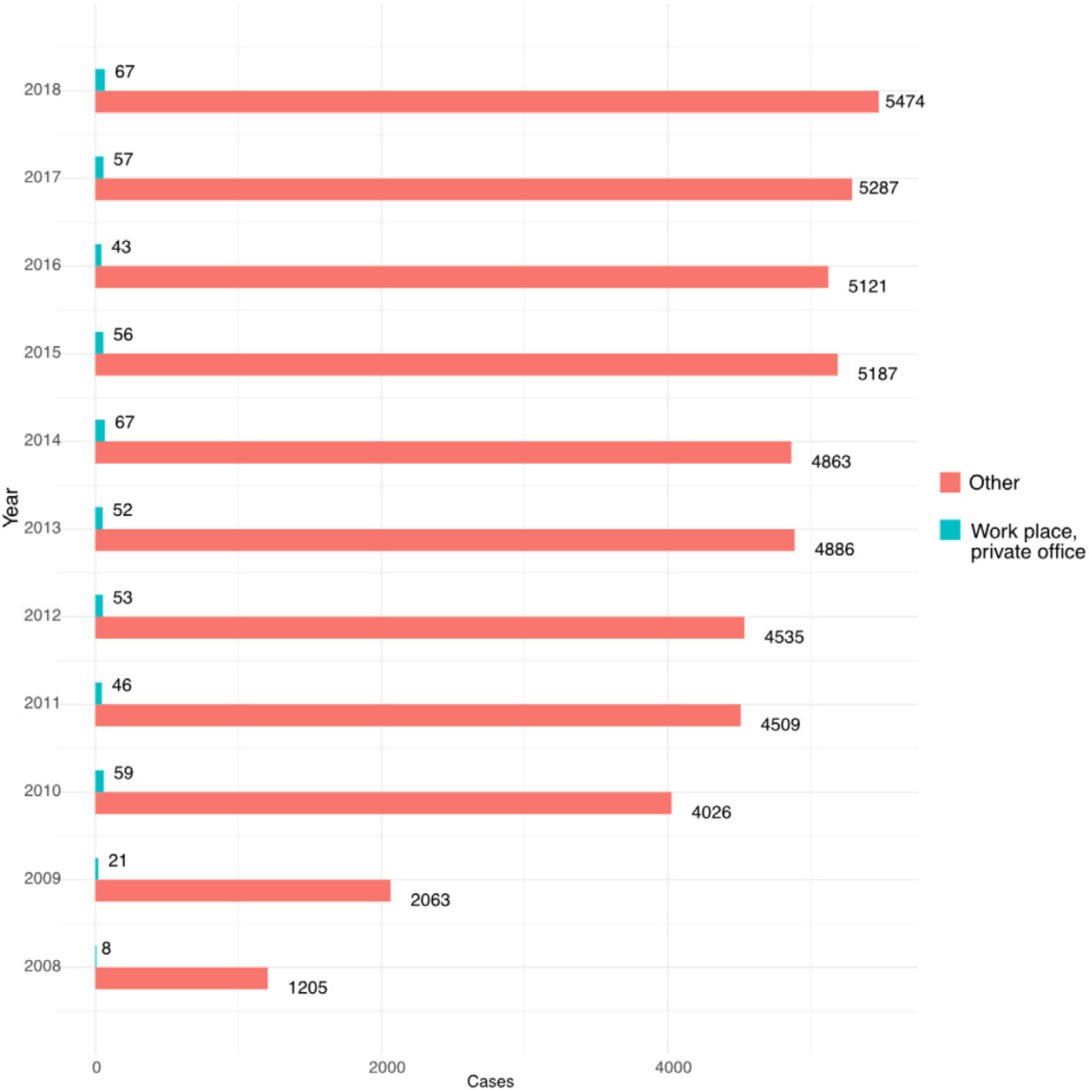
Incidence of out-of-hospital cardiac arrest at workplaces, 2008–2018. Incidence of out-of-hospital cardiac arrest (OHCA) at workplaces, 2008–2018: all patients included. Data is presented as cases in crude numbers for workplaces, including private office in relation to all other categories of places out of hospital (other), each year during the study period.

The incidence of OHCA at workplaces for each year, 2008–2018 remained low (Fig.5). All the OHCAs mostly occurred during the day or in the evening while the OHCA at workplaces most occurred during the day (Appendix, Figs. A2–A3).

## Discussion

The main findings in this nationwide registry-based study of patients with OHCAs were firstly that all locations of OHCA except for crowded public places, displayed significantly lower probability for survival than workplaces. Secondly, the patients with OHCAs at workplaces were more frequently defibrillated before the arrival of the EMS if they were found in a shockable rhythm, compared with all other groups. Thirdly, being found in a shockable rhythm was strongly associated with a higher chance of survival at workplaces.

The 30-day survival at workplaces was 30% and 22% after adjustment and this is far higher than the overall survival rate of 10.3% for OHCAs in Sweden in 2018.^25^ This is in line with a previous meta-analysis presenting a higher survival rate at workplaces compared with elsewhere,^14^ although no difference when compared with other public places. A study in Japan found an overall survival rate at workplaces of 5.8% for all cases, 10.3% for witnessed cases in 2006^15^ and 23.8% in a later study on bystander-witnessed OHCA of medical cause in 2020^17^ which was higher than at public places (16.2%). In a post-hoc analysis from Paris, which included 298 patients with OHCAs at different workplaces, the long-term survival, classified as survival with good neurological recovery at hospital discharge, was 13.4% and ranged from 0% to 23% at different workplaces.^16^ If we had categorised differently, we would have had a different survival rate since specific locations of OHCAs such as educational institutions (36.8%) and sports arenas (27.5%), for example, have reported a higher survival.^17^

This study shows that patients with OHCAs at workplaces were defibrillated more frequently before arrival of the EMS if they were found in a shockable rhythm (23%) than in all the other groups. Globally, bystander use of an AED after OHCA has been reported to range from 2% to 37%.^7^ Additionally, this study found that a shockable rhythm was associated with a greater chance of survival after OHCAs at workplaces. This is consistent with previous studies, but other factors, like bystander CPR, the use of PAD and the EMS response time, have also been reported to be associated with survival.^17^

We found that when private office was excluded, a lack of bystander CPR was associated with a lower chance of survival if OHCA occurred at workplaces. This finding make sense since if OHCA occurs in a private office, the potential rescuer may be found at a distance from the event and thereby will the start of bystander CPR be delayed. Moreover, female gender has previously been reported as a predictor of survival^27^ but recently of minor importance^28^ and our result, that female gender was associated with an increased chance of survival need to be supported in further studies. Adrenalin (Epinephrine) given has been shown to be associated with a lower survival after OHCA^29^ reflecting a prolonged resuscitation. The strong association between use of adrenalin and risk of death may be explained by confounding by indication.

Based on a relatively large sample size, we found that one per cent of patients with OHCAs in whom resuscitation was attempted experienced an OHCA at workplaces. The incidence was 10% in Japan^17^ and, in a meta-analysis from nine countries, the incidence ranged from 0.3%–4.7%.^14^ This variability may be explained by various definitions of workplaces.^14^, ^16^, ^17^ We included all types of office and industrial workplaces and it may be considered as a limitation.

The circumstances around OHCAs at workplaces differed from other places. The victims were younger with a more frequent cardiac aetiology. The arrest was mostly bystander witnessed, the rhythm was more often shockable with a more frequent defibrillation before arrival of the EMS. Thus, due to a number of factors, a relatively high survival rate can be expected. However, one in five victims of OHCAs at workplaces did not receive CPR before arrival of the EMS, as compared to one in four in all of Sweden and two of five in Europe.^3^ From a global perspective, the figure has varied between 20% and 80%.^7^

Adult BLS in Sweden includes training in CPR and AED with both compression and ventilation.^30^ If the bystander is unable to do ventilations the EMS dispatcher promotes chest compression only-CPR for adults. We do not know the reasons for the lack of bystander CPR and can only speculate about public fear of ventilation. Survival may increase with effective BLS and use of onsite AEDs.^11^, ^31^ Even if OHCAs mostly occur in residential settings, as in this study (70.9%), the time to defibrillation may decrease with more onsite AEDs. On the other hand, the AEDs, do not reach the patient in time^32^ but a mobile system for dispatching laypersons has been shown to increase bystander response.^33^

The median delay from collapse to defibrillation among patients who were found in a shockable rhythm at workplaces was 11 min. In a previous study, the median time from collapse to defibrillation was 5.8 min at workplaces.^17^ Thus, there is a potential for improvement. Practical applications for workplaces are to regularly “mass educate” the working public, which may benefit all locations and increase survival from OHCA.

## Limitations

(1) The classification of place of arrest may not be identical to the classification recommended by the Utstein style, but this classification is the most logical, based on existing data. (2) The categories of places are different from other performed studies and comparisons are therefore difficult to do. (3) The delays from collapse to treatments are based on estimations by bystanders and EMS personnel and all the reporting is by EMS personnel. (4) Information was missing for most of the variables. (5) Although the majority of patients suffering an OHCA in whom resuscitation was attempted are included in the register, a minority may not have been reported for logistical reasons.

## Conclusion

Out-of-hospital cardiac arrest occurring at workplaces and crowded public places display the highest probability of survival, as compared with other places outside hospital. An initial shockable cardiac rhythm was the strongest predictor of survival for OHCA at workplaces.

## Authors’ contributions

All the co-authors JH, AR, AC, ML, JL and HB, have contributed to the conception, design and the methodology of the study, analysed the results, critically revised the manuscript and have read and approved the final version of the manuscript to be submitted. HB was the main contributor to writing the manuscript. JH was the main supervisor. AR performed the formal statistical analysis. HB, AR and JH had full access to the data.

## Source of funding

This work was supported by the Swedish state under the agreement between the Swedish government and the County Council (the ALF agreement, ALFGBG 716901); the Foundation for Cardiopulmonary Resuscitation in Sweden; the Swedish Research Council (grant 2019-02019). All without any involvement in the study.

## Ethical approval

The Swedish Ethical Review Authority approved the ethical application on 28 October 2019 (2019-04066).

## Conflicts of interest

The authors declare that they have no conflicts of interest.

## Availability of data and materials

All the data analysed during this study are included in this article and its supplementary information files.

## References

[1] Berdowski J., Berg R.A., Tijssen J.G.P., Koster R.W. (2010). Global incidences of out-of-hospital cardiac arrest and survival rates: systematic review of 67 prospective studies. Resuscitation.

[2] Virani S.S., Alonso J.A., Benjamin S.E. (2020). Heart disease and stroke statistics—2020 update: a report from the American Heart Association. Circulation.

[3] Gräsner J.-T., Wnent J., Herlitz J. (2020). Survival after out-of-hospital cardiac arrest in Europe — results of the EuReCa TWO study. Resuscitation.

[4] Kiguchi T., Okubo M., Nishiyama C. (2020). Out-of-hospital cardiac arrest across the world: first report from the International Liaison Committee on Resuscitation (ILCOR). Resuscitation.

[5] Perkins G.D., Jacobs I.G., Nadkarni V.M. (2015). Cardiac arrest and cardiopulmonary resuscitation outcome reports: update of the Utstein Resuscitation Registry Templates for Out-of-Hospital Cardiac Arrest. Resuscitation.

[6] Perkins G.D., Jacobs I.G., Nadkarni V.M. (2015). Cardiac arrest and cardiopulmonary resuscitation outcome reports: update of the Utstein Resuscitation Registry Templates for Out-of-Hospital Cardiac Arrest. Circulation.

[7] Kiguchi T., Okubo M., Nishiyama C. (2020). Out-of-hospital cardiac arrest across the World: First report from the International Liaison Committee on Resuscitation (ILCOR). Resuscitation.

[8] Yan S., Gan Y., Jiang N. (2020). The global survival rate among adult out-of-hospital cardiac arrest patients who received cardiopulmonary resuscitation: a systematic review and meta-analysis. Crit Care.

[9] Weisfeldt M.L., Sitlani C.M., Ornato J.P. (2010). Survival after application of automatic external defibrillators before arrival of the emergency medical system: evaluation in the resuscitation outcomes consortium population of 21 million. J Am Coll Cardiol.

[10] Kitamura T., Iwami T., Kawamura T., Nagao K., Tanaka H., Hiraide A. (2010). Nationwide public-access defibrillation in Japan. N Engl J Med.

[11] Ringh M., Jonsson M., Nordberg P. (2015). Survival after Public Access Defibrillation in Stockholm, Sweden — a striking success. Resuscitation.

[12] Dyson K., Brown S.P., May S. (2019). International variation in survival after out-of-hospital cardiac arrest: a validation study of the Utstein template. Resuscitation.

[13] Fredman D., Ringh M., Svensson L. (2018). Experiences and outcome from the implementation of a national Swedish automated external defibrillator registry. Resuscitation.

[14] Descatha A., Dagrenat C., Cassan P., Jost D., Loeb T., Baer M. (2015). Cardiac arrest in the workplace and its outcome: a systematic review and meta-analysis. Resuscitation.

[15] Iwami T., Hiraide A., Nakanishi N. (2006). Outcome and characteristics of out-of-hospital cardiac arrest according to location of arrest: a report from a large-scale, population-based study in Osaka, Japan. Resuscitation.

[16] Palaghita A., Jost D., Despreaux T. (2016). Characteristics of cardiac arrest occurring in the workplace: a post hoc analysis of the Paris Area Fire Brigade Registry. J Occup Environ Med.

[17] Kobayashi D., Sado J., Kiyohara K. (2020). Public location and survival from out-of-hospital cardiac arrest in the public-access defibrillation era in Japan. J Cardiol.

[18] Descatha A., Jost D., Carpentier J.P. (2009). Is the workplace a site of cardiac arrest like any other?. Resuscitation.

[19] Swedish Work Environment Authority (2020).

[20] Rawshani A. (2020). The Swedish Registry of Cardiopulmonary Resuscitation — annual report 2020.

[21] Tjelmeland I.B.M., Masterson S., Herlitz J. (2020). Description of Emergency Medical Services, treatment of cardiac arrest patients and cardiac arrest registries in Europe. Scand J Trauma Resusc Emerg Med.

[22] Statistics Sweden. Statistics Sweden. 26 April 2020 ed. http://www.statistikdatabasen.scb.se/pxweb/en/ssd/START2020.

[23] Jacobs A.I., Nadkarni E.V., Bahr L.J. (2004). Cardiac arrest and cardiopulmonary resuscitation outcome reports: update and Simplification of the Utstein Templates for Resuscitation Registries A Statement for Healthcare Professionals From a Task Force of the International Liaison Committee on Resuscitation (American Heart Association, European Resuscitation Council, Australian Resuscitation Council, New Zealand Resuscitation Council, Heart and Stroke Foundation of Canada, InterAmerican Heart Foundation, Resuscitation Councils of Southern Africa). Circulation.

[24] Stromsoe A., Svensson L., Axelsson A.B., Goransson K., Todorova L., Herlitz J. (2013). Validity of reported data in the Swedish Cardiac Arrest Register in selected parts in Sweden (Report) (Author abstract). Resuscitation.

[25] Rawshani A. (2019). Swedish Registry of Cardiopulmonary Resuscitation (SRCR) — annual report 2019. https://hlrr.se/#hj%C3%A4rtstopp_utanf%C3%B6r_sjukhus.

[26] Wasserstein R.L., Lazar N.A. (2016). The ASA statement on p-values: context, process, and purpose. Am Stat.

[27] Adielsson A., Hollenberg J., Karlsson T. (2011). Increase in survival and bystander CPR in out-of-hospital shockable arrhythmia: bystander CPR and female gender are predictors of improved outcome. Experiences from Sweden in an 18-year perspective. Heart.

[28] Al-Dury N., Ravn-Fischer A., Hollenberg J. (2020). Identifying the relative importance of predictors of survival in out of hospital cardiac arrest: a machine learning study. Scand J Trauma Resusc Emerg Med.

[29] Holmberg M., Holmberg S., Herlitz J. (2002). Low chance of survival among patients requiring adrenaline (epinephrine) or intubation after out-of-hospital cardiac arrest in Sweden. Resuscitation.

[30] Greif R., Lockey A.S., Conaghan P. (2015). European Resuscitation Council Guidelines for Resuscitation 2015: Section 10. Education and implementation of resuscitation: Section 10. Education and implementation of resuscitation. Resuscitation.

[31] Valenzuela T.D., Roe D.J., Nichol G., Clark L.L., Spaite D.W., Hardman R.G. (2000). Outcomes of rapid defibrillation by security officers after cardiac arrest in casinos. N Engl J Med.

[32] Zijlstra J.A., Koster R.W., Blom M.T. (2018). Different defibrillation strategies in survivors after out-of-hospital cardiac arrest. Heart.

[33] Ringh M., Rosenqvist M., Hollenberg J. (2015). Mobile-phone dispatch of laypersons for CPR in out-of-hospital cardiac arrest. N Engl J Med.

