# Supplementary material for: Characteristics and outcome after out-of-hospital cardiac arrest with the emphasis on workplaces: an observational study from the Swedish Registry of Cardiopulmonary Resuscitation
Source: Resusc Plus. 2021 Feb 18;5:100090. doi: 10.1016/j.resplu.2021.100090 (PMC8244450; doi:10.1016/j.resplu.2021.100090)
Supplement: Supplementary file 1 [file mmc1.docx]

Supplemental data
*Characteristics and outcome after out-of-hospital cardiac arrest with the emphasis on workplaces: an observational study from the Swedish Registry of Cardiopulmonary Resuscitation, by Bylow et al.*

Supplemental, Appendix, Fig. A2 and Fig. A3. Time of the day, around the clock, for out-of-hospital cardiac arrest

Fig. A2. Time of the day, around the clock for out-of-hospital cardiac arrest (OHCA) during the study period 2008-20018
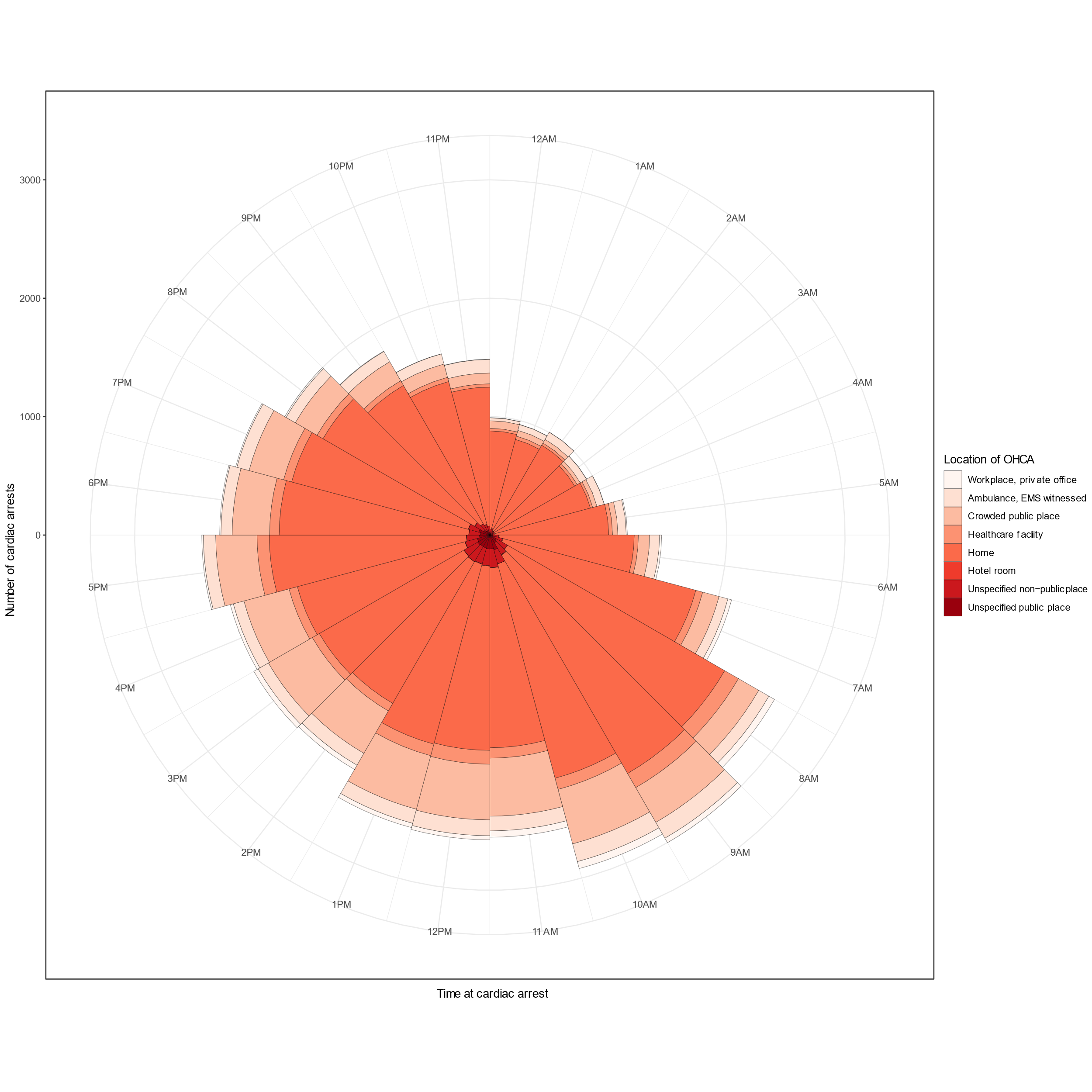


Figure legend Supplemental, Appendix, A2

Fig. A2. Time of the day presented around the clock, for out-of-hospital cardiac arrest (OHCA) during the study period 2008-20018. Time of the day for OHCA: all patient and locations included

Fig A3. Time of the day, around the clock, for out-of-hospital cardiac arrest, at workplaces
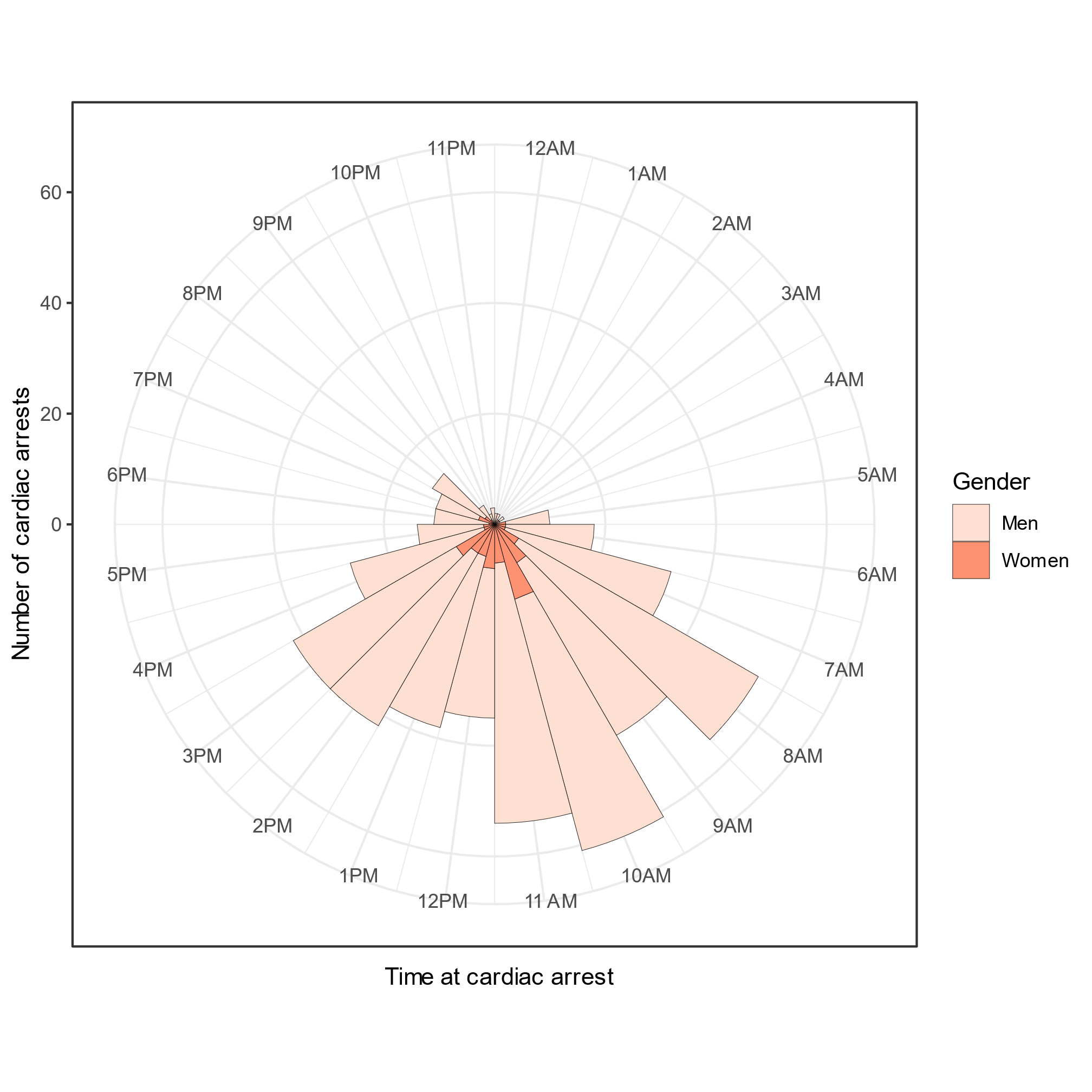


Figure legend Supplemental, Appendix, A3

Fig. A3. Time of the day, around the clock for out-of-hospital cardiac arrest at workplaces. Time of the day presented around the clock, for out-of-hospital cardiac arrest (OHCA) at workplaces during the study period 2008-20018: only patient at workplaces and private office included
