# Supplementary material for: Characteristics and outcome after out-of-hospital cardiac arrest with the emphasis on workplaces: an observational study from the Swedish Registry of Cardiopulmonary Resuscitation
Source: Resusc Plus. 2021 Feb 18;5:100090. doi: 10.1016/j.resplu.2021.100090 (PMC8244450; doi:10.1016/j.resplu.2021.100090)
Supplement: Supplementary file 2 [file mmc2.docx]

Supplemental data, Appendix, Table A1
*Characteristics and outcome after out-of-hospital cardiac arrest with the emphasis on workplaces: an observational study from the Swedish Registry of Cardiopulmonary Resuscitation, by Bylow et al.*

Appendix, Table A1

Supplemental data, missing values (a), all cases (b) and complete case analysis included in the regression model (c)

Table A1 a) Supplemental data with missing values

|  | **Workplace private office (N=529)** | **Ambulance -witnessed by EMS (N=2312)** | **Crowded public place (N=5844)** | **Healthcare  facility (N=1574)** | **Home, residential setting (N=33724)** | **Hotel room (N=75)** | **Un-specified non-public place (N=2074)** | **Un-specified public place (N=1553)** | **Overall (N=47685)** |
| --- | --- | --- | --- | --- | --- | --- | --- | --- | --- |
| **Location of arrest** |  |  |  |  |  |  |  |  |  |
| Workplace, private office | 529 (100%) | 0 (0%) | 0 (0%) | 0 (0%) | 0 (0%) | 0 (0%) | 0 (0%) | 0 (0%) | 529 (1.1%) |
| Ambulance -witnessed by EMS | 0 (0%) | 2312 (100%) | 0 (0%) | 0 (0%) | 0 (0%) | 0 (0%) | 0 (0%) | 0 (0%) | 2312 (4.8%) |
| Crowded public place | 0 (0%) | 0 (0%) | 5844 (100%) | 0 (0%) | 0 (0%) | 0 (0%) | 0 (0%) | 0 (0%) | 5844 (12.3%) |
| Healthcare facility | 0 (0%) | 0 (0%) | 0 (0%) | 1574 (100%) | 0 (0%) | 0 (0%) | 0 (0%) | 0 (0%) | 1574 (3.3%) |
| Home, residential setting | 0 (0%) | 0 (0%) | 0 (0%) | 0 (0%) | 33724 (100%) | 0 (0%) | 0 (0%) | 0 (0%) | 33724 (70.7%) |
| Hotel room | 0 (0%) | 0 (0%) | 0 (0%) | 0 (0%) | 0 (0%) | 75 (100%) | 0 (0%) | 0 (0%) | 75 (0.2%) |
| Un-specified non-public place | 0 (0%) | 0 (0%) | 0 (0%) | 0 (0%) | 0 (0%) | 0 (0%) | 2074 (100%) | 0 (0%) | 2074 (4.3%) |
| Un-specified public place | 0 (0%) | 0 (0%) | 0 (0%) | 0 (0%) | 0 (0%) | 0 (0%) | 0 (0%) | 1553 (100%) | 1553 (3.3%) |
| **Age** |  |  |  |  |  |  |  |  |  |
| Mean (SD) | 55.9 (12.5) | 72.0 (13.7) | 64.4 (16.5) | 75.2 (16.1) | 70.4 (15.7) | 62.2 (17.9) | 61.1 (17.7) | 65.8 (15.8) | 69.2 (16.1) |
| Median [Min, Max] | 58.0 [18.0, 93.0] | 73.0 [18.0, 100] | 67.0 [18.0, 97.0] | 80.0 [18.0, 104] | 73.0 [18.0, 106] | 62.0 [21.0, 92.0] | 64.0 [18.0, 98.0] | 68.0 [18.0, 97.0] | 72.0 [18.0, 106] |
| **Sex** |  |  |  |  |  |  |  |  |  |
| Men | 449 (84.9%) | 1417 (61.3%) | 4745 (81.2%) | 856 (54.4%) | 21306 (63.2%) | 59 (78.7%) | 1634 (78.8%) | 1243 (80.0%) | 31709 (66.5%) |
| Women | 80 (15.1%) | 895 (38.7%) | 1099 (18.8%) | 718 (45.6%) | 12412 (36.8%) | 16 (21.3%) | 440 (21.2%) | 310 (20.0%) | 15970 (33.5%) |
| Missing | 0 (0%) | 0 (0%) | 0 (0%) | 0 (0%) | 6 (0.0%) | 0 (0%) | 0 (0%) | 0 (0%) | 6 (0.0%) |
| **Witnessed arrest** |  |  |  |  |  |  |  |  |  |
| Not witnessed | 159 (30.1%) | 7 (0.3%) | 1602 (27.4%) | 365 (23.2%) | 12323 (36.5%) | 29 (38.7%) | 770 (37.1%) | 470 (30.3%) | 15725 (33.0%) |
| Witnessed | 360 (68.1%) | 2299 (99.4%) | 4042 (69.2%) | 1181 (75.0%) | 20659 (61.3%) | 45 (60.0%) | 1260 (60.8%) | 1032 (66.5%) | 30878 (64.8%) |
| Missing | 10 (1.9%) | 6 (0.3%) | 200 (3.4%) | 28 (1.8%) | 742 (2.2%) | 1 (1.3%) | 44 (2.1%) | 51 (3.3%) | 1082 (2.3%) |
| **Bystander** |  |  |  |  |  |  |  |  |  |
| Bystander CPR | 418 (79.0%) | 42 (1.8%) | 4255 (72.8%) | 1111 (70.6%) | 19686 (58.4%) | 46 (61.3%) | 1504 (72.5%) | 1074 (69.2%) | 28136 (59.0%) |
| No bystander CPR | 105 (19.8%) | 2040 (88.2%) | 1542 (26.4%) | 429 (27.3%) | 13077 (38.8%) | 19 (25.3%) | 557 (26.9%) | 458 (29.5%) | 18227 (38.2%) |
| Missing | 6 (1.1%) | 230 (9.9%) | 47 (0.8%) | 34 (2.2%) | 961 (2.8%) | 10 (13.3%) | 13 (0.6%) | 21 (1.4%) | 1322 (2.8%) |
| **Initial rhythm** |  |  |  |  |  |  |  |  |  |
| Not VF/VT | 272 (51.4%) | 1419 (61.4%) | 3337 (57.1%) | 1323 (84.1%) | 26865 (79.7%) | 55 (73.3%) | 1425 (68.7%) | 1016 (65.4%) | 35712 (74.9%) |
| VF/VT | 237 (44.8%) | 682 (29.5%) | 2276 (38.9%) | 173 (11.0%) | 5849 (17.3%) | 16 (21.3%) | 573 (27.6%) | 482 (31.0%) | 10288 (21.6%) |
| Missing | 20 (3.8%) | 211 (9.1%) | 231 (4.0%) | 78 (5.0%) | 1010 (3.0%) | 4 (5.3%) | 76 (3.7%) | 55 (3.5%) | 1685 (3.5%) |
| **Defibrillation** |  |  |  |  |  |  |  |  |  |
| Mean (SD) | 0.609 (0.488) | 0.429 (0.495) | 0.509 (0.500) | 0.208 (0.406) | 0.314 (0.464) | 0.406 (0.495) | 0.402 (0.491) | 0.444 (0.497) | 0.352 (0.478) |
| Median [Min, Max] | 1.00  [0, 1.00] | 0  [0, 1.00] | 1.00  [0, 1.00] | 0  [0, 1.00] | 0 [0, 1.00] | 0 [0, 1.00] | 0 [0, 1.00] | 0 [0, 1.00] | 0 [0, 1.00] |
| Missing | 17 (3.2%) | 58 (2.5%) | 145 (2.5%) | 86 (5.5%) | 1351 (4.0%) | 11 (14.7%) | 9 (0.4%) | 45 (2.9%) | 1722 (3.6%) |
| **Calendar year** |  |  |  |  |  |  |  |  |  |
| Mean (SD) | 2010 (2.83) | 2010 (2.86) | 2010 (2.83) | 2010 (2.77) | 2010 (2.84) | 2010 (2.84) | 2010 (2.91) | 2010 (2.89) | 2010 (2.85) |
| Median [Min, Max] | 2010 [2010, 2020] | 2010 [2010, 2020] | 2010 [2010, 2020] | 2010 [2010, 2020] | 2010 [2010, 2020] | 2010 [2010, 2020] | 2010 [2010, 2020] | 2010 [2010, 2020] | 2010 [2010, 2020] |
| **Adrenaline** |  |  |  |  |  |  |  |  |  |
| Adrenaline given | 415 (78.4%) | 1180 (51.0%) | 4264 (73.0%) | 1197 (76.0%) | 27629 (81.9%) | 54 (72.0%) | 1639 (79.0%) | 1202 (77.4%) | 37580 (78.8%) |
| Adrenaline not given | 104 (19.7%) | 1080 (46.7%) | 1526 (26.1%) | 360 (22.9%) | 5748 (17.0%) | 21 (28.0%) | 426 (20.5%) | 337 (21.7%) | 9602 (20.1%) |
| Missing | 10 (1.9%) | 52 (2.2%) | 54 (0.9%) | 17 (1.1%) | 347 (1.0%) | 0 (0%) | 9 (0.4%) | 14 (0.9%) | 503 (1.1%) |
| **Cause of arrest** |  |  |  |  |  |  |  |  |  |
| Heart disease | 343 (64.8%) | 1490 (64.4%) | 3635 (62.2%) | 871 (55.3%) | 20453 (60.6%) | 46 (61.3%) | 1122 (54.1%) | 950 (61.2%) | 28910 (60.6%) |
| Accident | 44 (8.3%) | 30 (1.3%) | 465 (8.0%) | 9 (0.6%) | 254 (0.8%) | 1 (1.3%) | 105 (5.1%) | 106 (6.8%) | 1014 (2.1%) |
| Drowning | 0 (0%) | 0 (0%) | 283 (4.8%) | 1 (0.1%) | 35 (0.1%) | 0 (0%) | 29 (1.4%) | 18 (1.2%) | 366 (0.8%) |
| Other | 94 (17.8%) | 471 (20.4%) | 832 (14.2%) | 341 (21.7%) | 6641 (19.7%) | 8 (10.7%) | 435 (21.0%) | 245 (15.8%) | 9067 (19.0%) |
| Overdose | 2 (0.4%) | 15 (0.6%) | 89 (1.5%) | 33 (2.1%) | 941 (2.8%) | 8 (10.7%) | 140 (6.8%) | 49 (3.2%) | 1277 (2.7%) |
| Pulmonary disease | 7 (1.3%) | 205 (8.9%) | 76 (1.3%) | 110 (7.0%) | 1956 (5.8%) | 2 (2.7%) | 47 (2.3%) | 23 (1.5%) | 2426 (5.1%) |
| Suffocation | 4 (0.8%) | 20 (0.9%) | 52 (0.9%) | 105 (6.7%) | 854 (2.5%) | 2 (2.7%) | 37 (1.8%) | 41 (2.6%) | 1115 (2.3%) |
| Suicide | 7 (1.3%) | 6 (0.3%) | 118 (2.0%) | 31 (2.0%) | 665 (2.0%) | 1 (1.3%) | 84 (4.1%) | 41 (2.6%) | 953 (2.0%) |
| Missing | 28 (5.3%) | 75 (3.2%) | 294 (5.0%) | 73 (4.6%) | 1925 (5.7%) | 7 (9.3%) | 75 (3.6%) | 80 (5.2%) | 2557 (5.4%) |
| **Cardiac aetiology** |  |  |  |  |  |  |  |  |  |
| Cardiac disease | 343 (64.8%) | 1490 (64.4%) | 3635 (62.2%) | 871 (55.3%) | 20453 (60.6%) | 46 (61.3%) | 1122 (54.1%) | 950 (61.2%) | 28910 (60.6%) |
| Non-cardiac aetiology | 158 (29.9%) | 747 (32.3%) | 1915 (32.8%) | 630 (40.0%) | 11346 (33.6%) | 22 (29.3%) | 877 (42.3%) | 523 (33.7%) | 16218 (34.0%) |
| Missing | 28 (5.3%) | 75 (3.2%) | 294 (5.0%) | 73 (4.6%) | 1925 (5.7%) | 7 (9.3%) | 75 (3.6%) | 80 (5.2%) | 2557 (5.4%) |

Table A1 b) Supplemental data of all cases

| All cases | Workplace,  private office | Ambulance -witnessed by EMS | Crowded public place | Healthcare  facility | Home, residential setting | Hotel room | Unspecified non-public place | Unspecified public place |
| --- | --- | --- | --- | --- | --- | --- | --- | --- |
| Total, n | 529 | 2312 | 5844 | 1574 | 33724 | 75 | 2074 | 1553 |
| Survival status,  n (%) | 157 (30.0) | 646 (28.0) | 1304 (22.7) | 142 (9.0) | 2307 (6.9) | 8 (11.0) | 337 (16.3) | 252 (16.5) |
| Age, years  (mean (SD)) | 55.91 (12.53) | 72.04 (13.70) | 64.37 (16.46) | 75.18 (16.13) | 70.44 (15.68) | 62.17 (17.85) | 61.08 (17.69) | 65.83 (15.85) |
| Sex, female, n (%) | 80 (15.1) | 895 (38.7) | 1099 (18.8) | 718 (45.6) | 12412 (36.8) | 16 (21.3) | 440 (21.2) | 310 (20.0) |
| Witnessed arrest, n (%) | 360 (69.4) | 2299 (99.7) | 4042 (71.6) | 1181 (76.4) | 20659 (62.6) | 45 (60.8) | 1260 (62.1) | 1032 (68.7) |
| No bystander CPR, n (%) | 105 (20.1) | 2040 (98.0) | 1542 (26.6) | 429 (27.9) | 13077 (39.9) | 19 (29.2) | 557 (27.0) | 458 (29.9) |
| Initial rhythm, VF/VT, n (%) | 237 (46.6) | 682 (32.5) | 2276 (40.5) | 173 (11.6) | 5849 (17.9) | 16 (22.5) | 573 (28.7) | 482 (32.2) |
| Defibrillation, n (%) | 312 (60.9) | 966 (42.9) | 2899 (50.9) | 310 (20.8) | 10176 (31.4) | 26 (40.6) | 831 (40.2) | 670 (44.4) |
| Calendar year,  n (%) |  |  |  |  |  |  |  |  |
| 2008 | 8 (1.5) | 83 (3.6) | 141 (2.4) | 28 (1.8) | 842 (2.5) | 1 (1.3) | 72 (3.5) | 38 (2.4) |
| 2009 | 21 (4.0) | 155 (6.7) | 283 (4.8) | 53 (3.4) | 1357 (4.0) | 4 (5.3) | 139 (6.7) | 72 (4.6) |
| 2010 | 59 (11.2) | 191 (8.3) | 495 (8.5) | 107 (6.8) | 2824 (8.4) | 6 (8.0) | 239 (11.5) | 164 (10.6) |
| 2011 | 46 (8.7) | 246 (10.6) | 552 (9.4) | 152 (9.7) | 3169 (9.4) | 9 (12.0) | 221 (10.7) | 160 (10.3) |
| 2012 | 53 (10.0) | 241 (10.4) | 567 (9.7) | 143 (9.1) | 3237 (9.6) | 13 (17.3) | 193 (9.3) | 141 (9.1) |
| 2013 | 52 (9.8) | 264 (11.4) | 654 (11.2) | 137 (8.7) | 3459 (10.3) | 8 (10.7) | 215 (10.4) | 149 (9.6) |
| 2014 | 67 (12.7) | 240 (10.4) | 626 (10.7) | 181 (11.5) | 3449 (10.2) | 6 (8.0) | 195 (9.4) | 166 (10.7) |
| 2015 | 56 (10.6) | 240 (10.4) | 637 (10.9) | 182 (11.6) | 3733 (11.1) | 7 (9.3) | 221 (10.7) | 167 (10.8) |
| 2016 | 43 (8.1) | 230 (9.9) | 621 (10.6) | 193 (12.3) | 3703 (11.0) | 5 (6.7) | 214 (10.3) | 155 (10.0) |
| 2017 | 57 (10.8) | 218 (9.4) | 617 (10.6) | 197 (12.5) | 3916 (11.6) | 6 (8.0) | 176 (8.5) | 157 (10.1) |
| 2018 | 67 (12.7) | 204 (8.8) | 651 (11.1) | 201 (12.8) | 4035 (12.0) | 10 (13.3) | 189 (9.1) | 184 (11.8) |
| Adrenaline not given, n (%) | 104 (20.0) | 1080 (47.8) | 1526 (26.4) | 360 (23.1) | 5748 (17.2) | 21 (28.0) | 426 (20.6) | 337 (21.9) |
| Cause of arrest, n (%) |  |  |  |  |  |  |  |  |
| Heart disease | 343 (68.5) | 1490 (66.6) | 3635 (65.5) | 871 (58.0) | 20453 (64.3) | 46 (67.6) | 1122 (56.1) | 950 (64.5) |
| Accident | 44 (8.8) | 30 (1.3) | 465 (8.4) | 9 (0.6) | 254 (0.8) | 1 (1.5) | 105 (5.3) | 106 (7.2) |
| Drowning | 0 (0.0) | 0 (0.0) | 283 (5.1) | 1 (0.1) | 35 (0.1) | 0 (0.0) | 29 (1.5) | 18 (1.2) |
| Other | 94 (18.8) | 471 (21.1) | 832 (15.0) | 341 (22.7) | 6641 (20.9) | 8 (11.8) | 435 (21.8) | 245 (16.6) |
| Overdose | 2 (0.4) | 15 (0.7) | 89 (1.6) | 33 (2.2) | 941 (3.0) | 8 (11.8) | 140 (7.0) | 49 (3.3) |
| Pulmonary disease | 7 (1.4) | 205 (9.2) | 76 (1.4) | 110 (7.3) | 1956 (6.2) | 2 (2.9) | 47 (2.4) | 23 (1.6) |
| Suffocation | 4 (0.8) | 20 (0.9) | 52 (0.9) | 105 (7.0) | 854 (2.7) | 2 (2.9) | 37 (1.9) | 41 (2.8) |
| Suicide | 7 (1.4) | 6 (0.3) | 118 (2.1) | 31 (2.1) | 665 (2.1) | 1 (1.5) | 84 (4.2) | 41 (2.8) |
| Non-cardiac aetiology, n (%) | 158 (31.5) | 747 (33.4) | 1915 (34.5) | 630 (42.0) | 11346 (35.7) | 22 (32.4) | 877 (43.9) | 523 (35.5) |

Table A1 c) Supplemental data of complete case analysis included in the regression model

| Complete case analysis included in the regression model | Workplace,  private office | Ambulance -witnessed by EMS | Crowded public place | Healthcare facility | Home, residential setting | Hotel room | Unspecified non-public place | Unspecified public place |
| --- | --- | --- | --- | --- | --- | --- | --- | --- |
| Total, n | 440 | 1820 | 4910 | 1312 | 28 957 | 55 | 1868 | 1311 |
| Survival status, n (%) | 123 (28.0) | 515 (28.3) | 1088 (22.2) | 99 (7.5) | 1896 (6.5) | 6 (10.9) | 279 (14.9) | 210 (16.0) |
| Age, years (mean (SD)) | 56.43 (12.13) | 72.02 (13.66) | 64.55 (16.30) | 75.07 (16.14) | 70.59 (15.56) | 59.27 (17.01) | 60.85 (17.69) | 66.44 (15.26) |
| Sex, female, n (%) | 62 (14.1) | 721 (39.6) | 901 (18.4) | 607 (46.3) | 10559 (36.5) | 11 (20.0) | 391 (20.9) | 266 (20.3) |
| Witnessed arrest, n (%) | 305 (69.3) | 1815 (99.7) | 3520 (71.7) | 994 (75.8) | 18307 (63.2) | 31 (56.4) | 1149 (61.5) | 901 (68.7) |
| No bystander CPR, n (%) | 82 (18.6) | 1782 (97.9) | 1309 (26.7) | 370 (28.2) | 11397 (39.4) | 14 (25.5) | 487 (26.1) | 377 (28.8) |
| Initial rhythm, VF/VT, n (%) | 207 (47.0) | 585 (32.1) | 2003 (40.8) | 149 (11.4) | 5341 (18.4) | 14 (25.5) | 532 (28.5) | 429 (32.7) |
| Defibrillation, n (%) | 270 (61.4) | 772 (42.4) | 2518 (51.3) | 277 (21.1) | 9218 (31.8) | 23 (41.8) | 760 (40.7) | 591 (45.1) |
| Calendar year, n (%) |  |  |  |  |  |  |  |  |
| 2008 | 7 (1.6) | 58 (3.2) | 121 (2.5) | 25 (1.9) | 715 (2.5) | 1 (1.8) | 71 (3.8) | 33 (2.5) |
| 2009 | 20 (4.5) | 111 (6.1) | 251 (5.1) | 45 (3.4) | 1238 (4.3) | 4 (7.3) | 125 (6.7) | 56 (4.3) |
| 2010 | 45 (10.2) | 151 (8.3) | 408 (8.3) | 88 (6.7) | 2451 (8.5) | 5 (9.1) | 195 (10.4) | 137 (10.5) |
| 2011 | 37 (8.4) | 188 (10.3) | 399 (8.1) | 108 (8.2) | 2494 (8.6) | 6 (10.9) | 191 (10.2) | 114 (8.7) |
| 2012 | 42 (9.5) | 193 (10.6) | 445 (9.1) | 111 (8.5) | 2619 (9.0) | 2 (3.6) | 171 (9.2) | 120 (9.2) |
| 2013 | 40 (9.1) | 183 (10.1) | 505 (10.3) | 97 (7.4) | 2699 (9.3) | 6 (10.9) | 189 (10.1) | 106 (8.1) |
| 2014 | 51 (11.6) | 171 (9.4) | 500 (10.2) | 123 (9.4) | 2795 (9.7) | 6 (10.9) | 173 (9.3) | 144 (11.0) |
| 2015 | 46 (10.5) | 178 (9.8) | 549 (11.2) | 159 (12.1) | 3364 (11.6) | 7 (12.7) | 207 (11.1) | 143 (10.9) |
| 2016 | 39 (8.9) | 205 (11.3) | 558 (11.4) | 186 (14.2) | 3445 (11.9) | 5 (9.1) | 199 (10.7) | 137 (10.5) |
| 2017 | 52 (11.8) | 198 (10.9) | 572 (11.6) | 183 (13.9) | 3488 (12.0) | 5 (9.1) | 167 (8.9) | 146 (11.1) |
| 2018 | 61 (13.9) | 184 (10.1) | 602 (12.3) | 187 (14.3) | 3649 (12.6) | 8 (14.5) | 180 (9.6) | 175 (13.3) |
| Adrenaline not given, n (%) | 84 (19.1) | 840 (46.2) | 1226 (25.0) | 271 (20.7) | 4558 (15.7) | 10 (18.2) | 345 (18.5) | 275 (21.0) |
| Cause of arrest, n (%) |  |  |  |  |  |  |  |  |
| Heart disease | 308 (70.0) | 1205 (66.2) | 3273 (66.7) | 759 (57.9) | 18690 (64.5) | 38 (69.1) | 1064 (57.0) | 870 (66.4) |
| Accident | 33 (7.5) | 24 (1.3) | 399 (8.1) | 9 (0.7) | 234 (0.8) | 0 (0.0) | 95 (5.1) | 89 (6.8) |
| Drowning | 0 (0.0) | 0 (0.0) | 230 (4.7) | 1 (0.1) | 32 (0.1) | 0 (0.0) | 27 (1.4) | 13 (1.0) |
| Other | 81 (18.4) | 395 (21.7) | 720 (14.7) | 300 (22.9) | 6078 (21.0) | 7 (12.7) | 395 (21.1) | 219 (16.7) |
| Overdose | 1 (0.2) | 10 (0.5) | 70 (1.4) | 27 (2.1) | 813 (2.8) | 7 (12.7) | 131 (7.0) | 34 (2.6) |
| Pulmonary disease | 7 (1.6) | 166 (9.1) | 70 (1.4) | 97 (7.4) | 1749 (6.0) | 0 (0.0) | 44 (2.4) | 20 (1.5) |
| Suffocation | 4 (0.9) | 16 (0.9) | 44 (0.9) | 91 (6.9) | 771 (2.7) | 2 (3.6) | 34 (1.8) | 34 (2.6) |
| Suicide | 6 (1.4) | 4 (0.2) | 104 (2.1) | 28 (2.1) | 590 (2.0) | 1 (1.8) | 78 (4.2) | 32 (2.4) |
| Non-cardiac aetiology, n %) | 132 (30.0) | 615 (33.8) | 1637 (33.3) | 553 (42.1) | 10267 (35.5) | 17 (30.9) | 804 (43.0) | 441 (33.6) |
