# Supplementary figures and images for: Characteristics and outcome after out-of-hospital cardiac arrest with the emphasis on workplaces: an observational study from the Swedish Registry of Cardiopulmonary Resuscitation
Source: Resusc Plus. 2021 Feb 18;5:100090. doi: 10.1016/j.resplu.2021.100090 (PMC8244450; doi:10.1016/j.resplu.2021.100090)

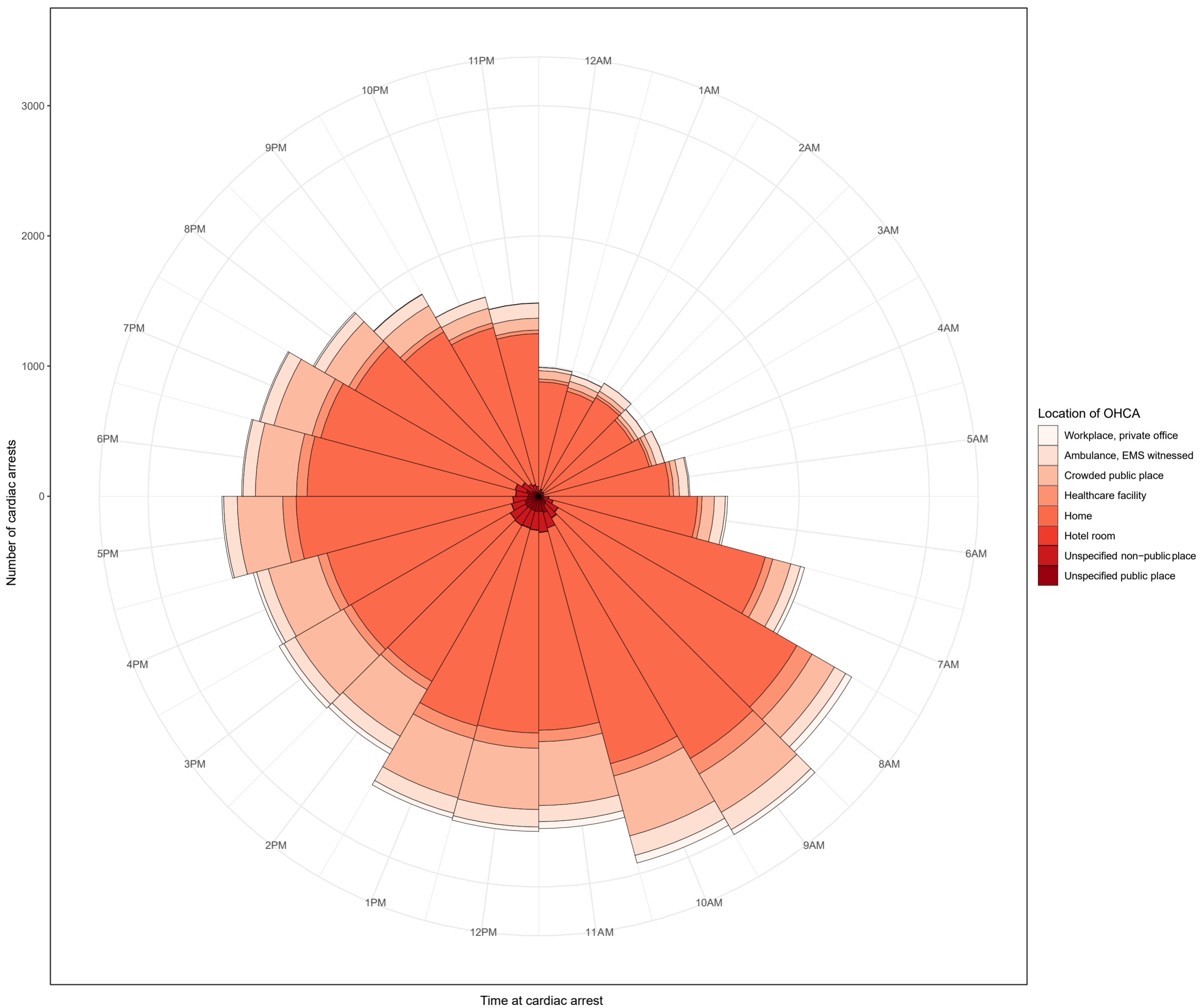

Supplement: Supplementary file 3 [file mmc3.pdf]

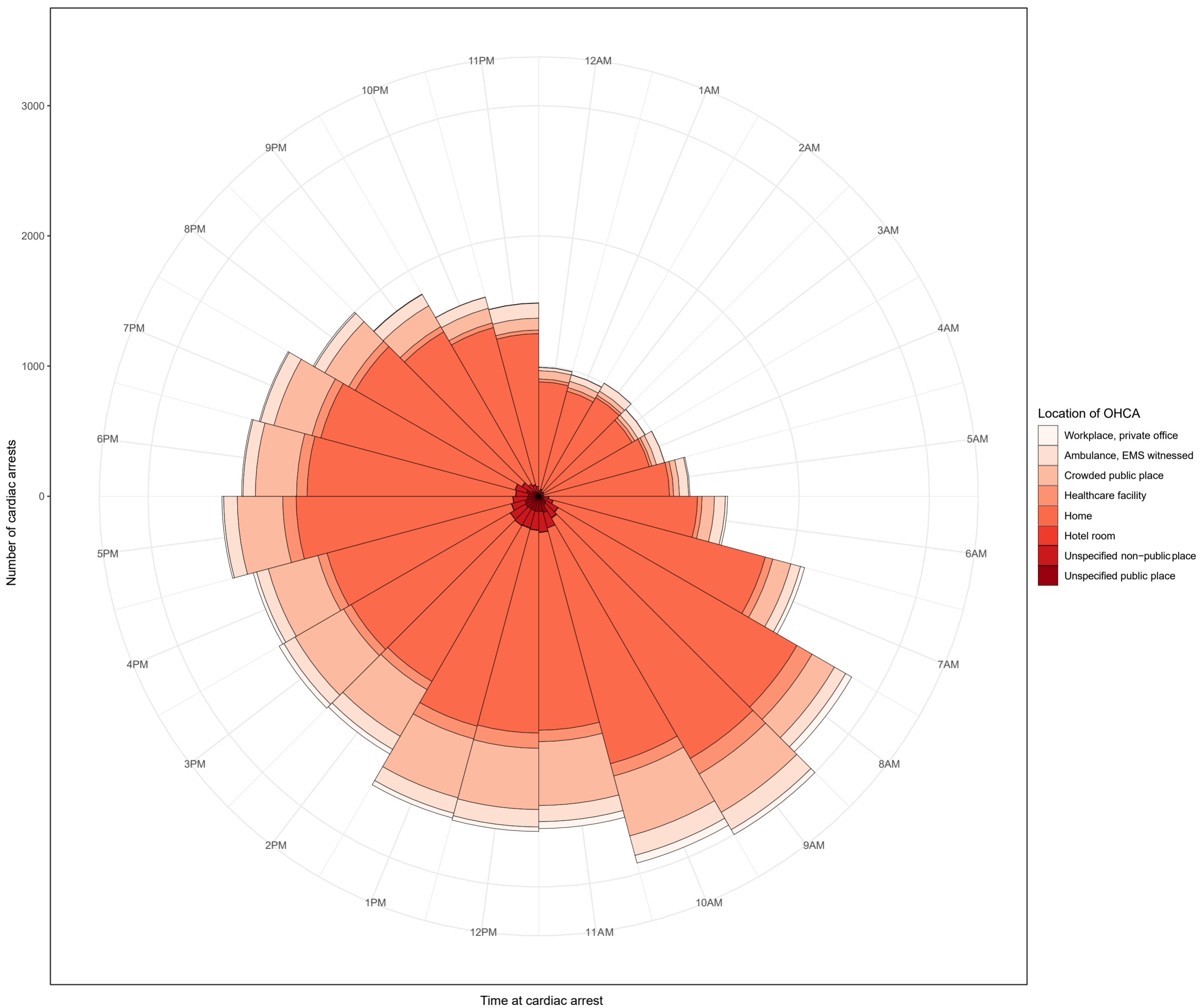

Supplement: Supplementary file 4 [file mmc4.pdf]

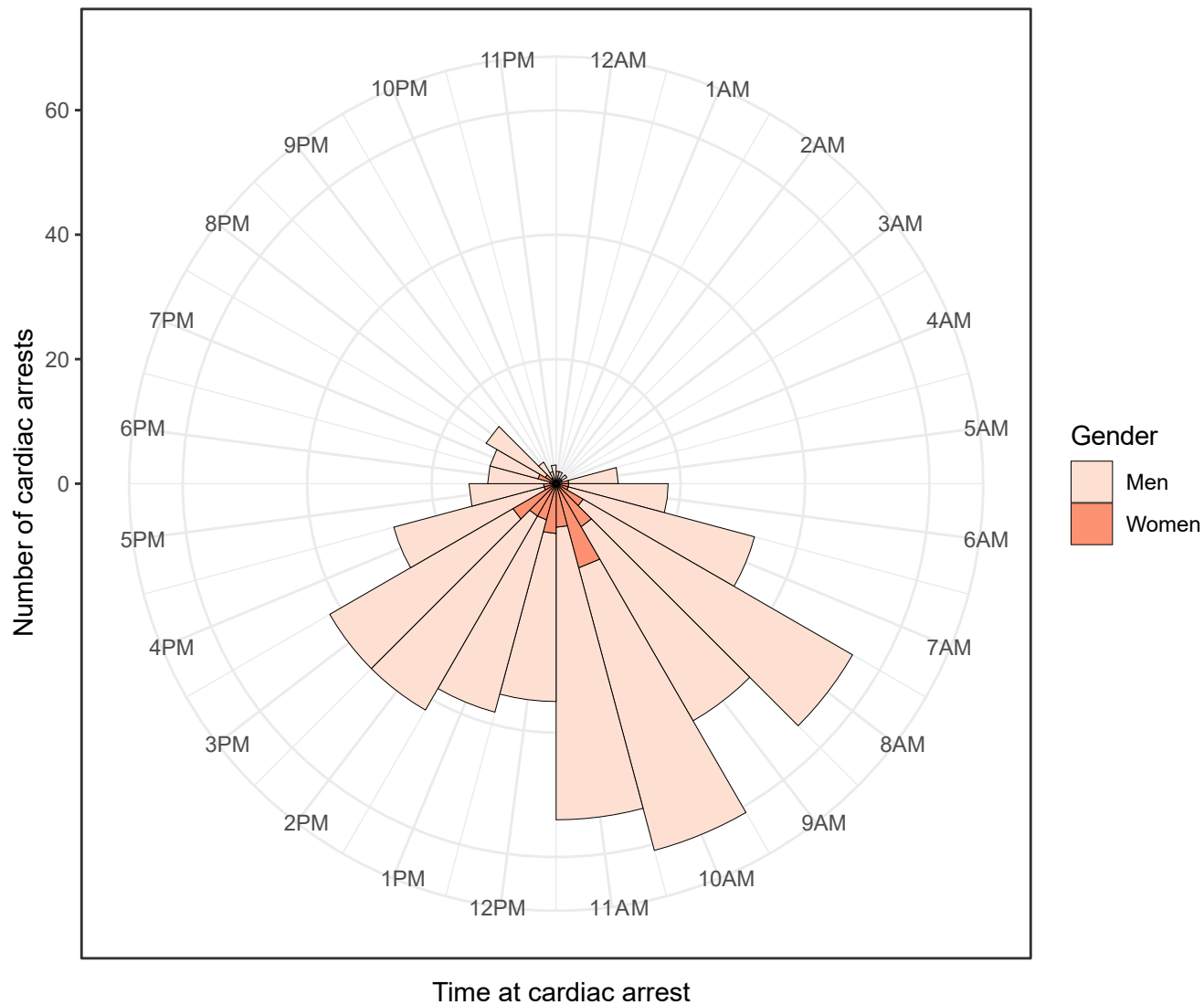

Supplement: Supplementary file 5 [file mmc5.pdf]
